# Health system structure and its influence on outcomes: The Canadian experience

**DOI:** 10.1177/08404704241248559

**Published:** 2024-05-13

**Authors:** Braden J. Manns, Stephanie Hastings, Greg Marchildon, Tom Noseworthy

**Affiliations:** 1 2129 University of Calgary, Calgary, Alberta, Canada.; 2 274071 University of Toronto, Toronto, Ontario, Canada.

## Abstract

Healthcare delivery systems in Canada are structured using three models: individual institutions, health regions, and single provincial systems, usually with smaller geographic zones. The comparative ability of these models to improve care, outcomes, and the Quadruple Aim is largely unstudied. We reviewed Canadian studies examining outcomes of provincial healthcare delivery system restructuring. Across models, results were inconsistent, and quality of evidence was low. For all provinces, primary care sits outside healthcare delivery systems, with limited governance and integration. The single provincial model can reduce costs of non-clinical support functions like finance, human resources, and analytics. This model may also be best at reducing variations in care, improving electronic information integration that enables clinical decision support and reporting, and supporting the provincial spread and scale of innovations, but further refinements are required and existing studies have major limitations, limiting definitive conclusions.

## Background

Healthcare structures and organizations in Canada are undergoing unprecedented change. In 2023, Alberta announced that its centralized integrated healthcare delivery system was being split into three separate organizations: acute care, mental health and addiction, and continuing care, with a new primary care organization being added. Also in 2023, Newfoundland and Labrador transitioned from health regions to a centralized health authority, and Quebec passed legislation to do the same. In 2019, Ontario announced plans to create Ontario Health, including 57 Ontario Health Teams, though progress has been slow.

At the federal level, the Canadian government is responsible for setting national standards for Medicare and provides healthcare services for Indigenous people living on reserve and members of the Canadian Forces. At the provincial level, Canada’s healthcare system has macro-, meso-, and micro-level structures. At the macro level, the functions of the health ministries and the roles of provincial governments have mostly remained static. They are responsible for legislation, funding, and policy direction, for negotiating and managing contracts with doctors, and negotiating salaries with unionized staff. Provincial health ministries do not deliver healthcare. They delegate this authority to administrative bodies at the meso level.

At the meso level—the healthcare delivery structures—large-scale changes have occurred frequently over the past 30 years. Until the 1980s, hospitals in Canada functioned independently, each governed by its own management structure and board. Provincial governments served primarily as funders for hospitals, and separately for physicians. All provinces, except Ontario, moved to health regions between 1989 and 1996, where a province is split into regions based on geography and referral patterns, with a focus on caring for a defined population and offering healthcare services based on population needs. The theoretical benefits of such health regions are better integration of in-patient and out-patient services, reduced costs, improved quality and consistency of care, greater focus on population health, increased accountability, more public engagement, and better matching of resources to local needs^1^ (Table 1). Several provinces have since moved to more centralized structures, often with zones, that should theoretically have similar benefits to health regions and could have added benefits (Table 1).

**Table 1.** Rationale for different healthcare delivery models in Canada (based on Marchildon, 2016).

Targeted improvement	Example
*Ontario’s Health Teams (vs. independent hospital model)*	
Vertical integration: Integrate and coordinate a broad range of health services	Improving service integration through better networking of care providers accountable for the delivery of care to a defined patient population
Decrease variation and improve service quality through evidence-based practice	Benchmarking quality of care according to a set of key indicators
Increase accountability by having health teams report on performance and outcomes to the health ministry	Making the Ontario Health Teams (and their network of providers) accountable for achieving the key outcomes defined
*Health region (vs. independent hospital model)*	
Decrease variation and improve service quality through evidence-based practice	Reduce care variation through common regional guidelines and care pathways; focus on quality across the system, not just in each silo
Vertical integration: Integrate and coordinate the range of health services	Improving service integration through better networking of care providers accountable for care delivery to a defined population
Population health: Shift emphasis and resources to health promotion	Move system focus to prevention (rather than treating illness) by moving away from an exclusive focus on hospitals
Horizontal integration: Consolidate and rationalize services to reduce costs	Reduce healthcare costs by focusing on over and underuse of effective therapies
Decentralize decision-making to increase public participation and input	Increase public participation through creation of regional health advisory committees
Increase accountability by having health regions report on performance and outcomes to the health ministry	Improve accountability for costs and outcomes, since performance and outcomes reporting is easier at a larger scale
Decentralize resources to facilitate better match with population needs	Funding provided to health regions which is allocated to services that meet local healthcare needs
*Single provincial model (vs. health region)*	
Decrease variation and improve service quality through evidence-based practice	Reduce variation in care by creating provincial guidelines and care pathways), improving quality of care provincially through improved ability to spread and scale innovation^2,3^
Horizontal integration: Consolidate and rationalize services to reduce costs	Reducing administrative costs, and overall healthcare costs by benchmarking similar service areas
Increase accountability by having the single delivery system report performance and outcomes to the health ministry	Improve accountability for costs and outcomes, since performance and outcomes reporting could be easier with a single electronic medical record and data and analytics team
Amalgamating non-clinical functions	Central procurement, information technology, data and analytics, research, legal, finance, and human resources
Electronic integration	Enabling electronic integration, through provincial implementation of a common electronic health record

At the micro level, the role of hospitals and physicians has also been relatively stable. Regardless of the payment model, physicians have largely remained independent contractors with a payment relationship to the Minister of Health, bypassing the meso level. In all provinces, there has been limited integration of primary care with the rest of the healthcare delivery system, and a lack of formal primary care governance.^4^

In this article, we review the current state of each province’s healthcare delivery structure and examine what the published literature tells us about the impact of different healthcare delivery structures on outcomes, including the Quadruple Aim (i.e., patient experience, population health [including patient outcomes], healthcare provider experience, and value for money).^5^

## Methods

The authors searched government and healthcare delivery system web sites, published literature, and national news articles to identify current delivery systems and their governance models, use of centralized non-clinical functions, provincial structures for scale and spread of innovations, and primary care organizational arrangements. Where needed, and when a member of our team was unable to confirm the accuracy of the information, we contacted provincial experts (provincial or regional health system leaders, provincial health ministry staff, and academic researchers) for validation.

To determine the benefits and limitations of different healthcare delivery structures on the Quadruple Aim, we updated a 2016 scoping review by Bergevin et al.^6^ We searched for papers and reports examining outcomes associated with structural changes in Canada using keywords such as regionalization, centralization, and restructuring in combination with health and Canada or individual provinces. We also checked reference lists of included papers for relevant titles. We limited our analysis to those papers reporting quantitative or qualitative data to compare systems before and after restructuring, or between provinces. Reviews, news articles, and opinion pieces not reporting quantitative or qualitative data were excluded from analysis. We extracted outcomes from eligible studies based on the framework in Table 1 and expectations of regionalization.^1^

We assessed study quality using the Mixed Methods Appraisal Tool (MMAT^7^), which includes two screening questions for all study types and five design-dependent quality criteria questions. All studies meeting the eligibility requirements noted above were extracted regardless of study quality. Data was extracted by one author, with the accuracy of data abstraction confirmed by a second author.

## Results

### Current healthcare delivery system structures

Of the nine provinces which established Regional Health Authorities (RHAs) in the early to mid-1990s, six now have or are moving towards a provincial healthcare delivery system model, first started in 2008 in Alberta. Provinces with a single delivery system (except Prince Edward Island (PEI)) have smaller geographic zones (“areas” in Saskatchewan), serving similar functions to RHAs but without as much autonomy. There is some variation in the roles, responsibilities, and autonomy of zones, but each is knit together by and reports to an overarching governance body that sits outside of the ministry (Table 2).

**Table 2.** Current healthcare delivery system structures.

Model	Accountable organization	Regional structure			Provincial structure					Health sector organization model
Province and year of most recent change	Ontario 2019	British Columbia 2001	Manitoba 2012	New Brunswick 2008	Newfoundland and Labrador 2023	Nova Scotia 2015	Prince Edward Island 2010	Québec currently in transition	Saskatchewan 2017	Alberta currently in transition
Structure description	Ontario Health oversees healthcare planning and delivery LHINs have been replaced by Ontario Health57 Ontario Health Teams (OHTs) at varying stages of readiness are meant to facilitate care planning and integration for a defined geographic population	Five regional health authorities are funded to deliver health servicesProvincial Health Services Authority works in partnership with regional authorities to provide highly specialized servicesThe First Nations Health Authority plans and delivers some healthcare services for First Nations	Five regional health authoritiesShared Health Services works with the regional authorities to plan and implement a coordinated provincial approach	Two regional health authorities	Newfoundland and Labrador Health Services responsible for healthcare planning and delivery, working with five zones	Nova Scotia Health Authority, and its four NSHA zones, operates hospitals, health centres, and community-based programs across the provinceNSHA is partnered with IWK Health (hospital focused on women’s and children’s health); which has own boardTajikeimik (Mi’kmaw Health and Wellness) is in development to partner with NSHA to design and deliver health services for First Nations	Health PEI responsible for healthcare delivery and planningNo regional substructure	Currently there are 34 regional health entities (22 known as CISSS (integrated health and social services centre) or CIUSSS (integrated university health and social services centre) plus other hospitals or service providersIn Dec 2023, Bill 15 established a provincial health authority, Sante Quebec, which will integrate and oversee these systems and provide both health and social services in a single organization	Saskatchewan Health Authority, with six geographic areas responsible for acute, continuing, and some primary care clinics and planning in their respective regions	Four Health sector organization model announced in 2023: Primary Care Organization will be newly created; Alberta Health Services (integrated province-wide health system since 2008) will be split into an Acute Care organization, Mental Health and Addictions organization, and Continuing Care organization
Governance	Board of directors for Ontario HealthOHT governance will vary based on team structureHospitals will continue as independent corporations run by their own board of directors	Each health authority has board of directors	Each RHA has a board of directors	Each RHS has a board of directors	Board of trustees for Newfoundland and Labrador Health ServicesZone councils focus on specific regional issues, will advise the provincial health authority	Single NSHA administrator appointed in 2021 after dissolution of boardBoard of directors for IWK	Board of directors for Health PEI	Sante Quebec will have own board of directorsEach region currently has own board of directors	Board of directors for Saskatchewan Health Authority	Alberta Ministry of Health will provide enhanced oversight. AHS will maintain a board, with tighter links to Ministry. The 4 organizations will report to an Overarching Integration Council led by Health Minister
Central procurement	In planning	✓	✓	✓	✓	✓	✓	X	✓	AHS had central procurement
Common IT	X	X	✓	✓	✓	✓	✓	X	✓	AHS had common IT
Single electronic health record for hospitals	X	X	X	X	In planning	In planning	✓	In planning	X	✓
Provincial structure for scale and spread	X	✓	X	✓	In planning	✓	X	X	In planning	✓
Primary care governance overall and multidisciplinary team funding	Overall: no formal overarching governance for primary care	Overall: no formal overarching governance for primary care	Overall: no formal overarching governance for primary care	Overall: no formal overarching governance for primary care	Overall: no formal overarching governance for primary care	Overall: no formal overarching governance for primary care	Overall: no formal overarching governance for primary care	Overall: no formal overarching governance for primary care	Overall: no formal overarching governance for primary care	Primary Care Organization will oversee primary care; details not yet available
	Family Health Teams (n = 184) receive government funding for multidisciplinary team members, each with its own board	Practices can receive up to $15,000 per FTE through Family Practice Services Committee	My Health Teams are voluntary teams of healthcare providers working together to plan and deliver care for geographic area or specific community or population. Governance varies; teams receive funding based on meeting patient attachment goals ($52,500 - $75,000 per year reported as of 2013)	No funding noted for multidisciplinary teams	Family Practice Networks are voluntary non-profit corporations organizing participation of physicians to work with their regions to identify and solve unmet needs at the community levelGovernment funded Family Care Teams are being created over the next five years. Funding unclear but family physicians have been hired on salary to FCTs	Collaborative Family Practice Teams (n = 99) are fully or partly funded by Nova Scotia Health	Five Primary Care Networks, which are voluntary to joinPatient medical homes are being developed and will receive government funding for team-based care	Family Medicine Groups (FMGs) are small groups of physicians working with allied health. Super-clinics are FMGs that meet criteria determined by government (opening hours, access requirements) and receive additional funding to cover a larger populationFMGs receive funding for nursing staff based on number of patients enrolled (approximately 2 nurses per 15,000 enrolled patients)	35 Health Networks have been created based on geographic needs to enable team-based care in the community with some funding for allied health	Primary Care Networks receive $62 per rostered patient for multidisciplinary team members, governance is provided through a Provincial PCN Committee and five zone PCN committees

Despite five provinces following Alberta’s lead, Alberta is now moving from a single provincial model to a unique form, where service delivery will be administered by four separate public organizations.

Ontario stands out as it continues with a system with independent hospitals and hospital boards. It has never had health regions directly responsible for service delivery, though it used Local Health Integration Networks (LHINs) between 2006 and 2019 to facilitate the integration of services. In 2019, Ontario created Ontario Health, which amalgamated six provincial agencies and the 14 LHINs into one central agency. Ontario Health has begun to delegate most meso-level governance and decision-making to 57 Ontario Health Teams (OHTs) comprising independent hospitals; primary care practices; and rehabilitation, palliative, home, and community care organizations.

### Impact of healthcare delivery structures on the Quadruple Aim

We identified seven quantitative studies across three provinces, examining over 20 outcomes. Six were pre-post comparisons and one was a cross-provincial comparison. All quantitative studies were of low quality, usually because of poor descriptions of methodology, inadequate justification of measures selected for comparison, lack of appropriate controls, and no significance testing. No studies had rigorous controlled designs. We also identified two qualitative studies involving interviews with health system leaders that were of medium quality.

There was limited evidence on how healthcare delivery structure influences the Quadruple Aim (Table 3). Only Bergevin et al.^6^ examined the impact of structure on staff experience, and results were mixed.

**Table 3.** Outcomes of health system delivery structure changes.

Author (year) and structure under study	Study design and quality	Integration and coordination of services (vertical integration)	Consolidation and rationalization of services to reduce costs (horizontal integration)	Shift to health promotion	Decrease variation and improve quality	Match resource use with population needs	Public participation	Increased accountability
*RHAs vs. local structures*								
Bergevin et al. (2016)^a^BC, MB, NB, NL, QC, SK pre- and post-creation of respective regional health authorities	Qualitative analysis of interviews with ∼3 senior leaders in each province **Medium quality**	+ BC, NL, and QC	+ rationalization of services BC, NL, and QC + costs BC, MB, NB costs SK+ economies of scale QC and SK− significant cost of restructuring QC and SK	− health promotion effectiveness BC+ shift of funding to community-based care BC, MB, NL, and QC+ focus on population health MB, NB, NL, QC, and SK	organizational capacity SKvariability across regions in ability to make evidence-based decisions QC+ standardizing best practices NB	+ BC, MB, NL, and QC	+ MB, NB, NL, and QC	
*Provincial vs. RHAs*								
Bergevin et al. (2016) AB, NS, and PEI pre- and post-creation of provincial health authorities	Qualitative analysis of interviews with senior leaders **Medium quality**	+ AB	+ rationalization of services AB and PEI/any cost savings not attributable to structure AB− no evidence of cost savings; significant costs of restructuring NS	+ AB				+ expanded information system and increased analytic and evaluative capacity PEI+ efforts to standardize data formats and information systems NS
Donaldson (2010) Alberta pre- and post-merger to AHS	Cohort study using CIHI data (2006 vs. 2009), includes other provinces for comparison **Low quality**				hip, knee, and bypass wait times			
Duckett (2011) Alberta pre- and post-merger to AHS	Before and after study using senior management salary data (2007/08 vs. 2009/10) **Low quality**		+ management salary					
Fierlbeck (2019) Nova Scotia pre- and post-merger to NSHA	Before and after study using CIHI data (2013 vs. 2018) **Low quality**		− total spending					
			/admin costs					

*Ontario Local Health Integration Networks (LHINs) vs Ontario before LHINs*								
Auditor General of Ontario (2015)	Audit involving mixed methods: surveys, document reviews, before and after study using Ministry of Health and other reports (2007/09/10 vs. 2015) **Low quality**		Mixed interview results on economic efficiencies brought by LHINs		+ ED LOS,^b^ MRI scan and cancer surgery within guidelines, wait time for in-home community services^c^			Many LHINs not tracking performance or quality; Ministry takes little or inconsistent action on low performance
Ontario pre- and post-LHIN creation					cardiac bypass procedures within guidelines)			
					− (Readmissions, % ALC days, repeat unplanned ED visits;^d^ inequities across province in care and access to services)			
Barker (2021)	Before and after study using Health Quality Ontario, Ministry of Finance, and other reports (2008/09/10 vs. 2015/16/17 based on data availability) **Low quality**		+^e^	+	+ avoidable hospitalizations		+^f^	
Ontario pre- and post-LHIN creation					ED LOS, discharged patients who saw physician within 70 days			
					− unplanned readmissions			
Bergevin et al. (2016)	Qualitative analysis of interviews with ∼3 senior leaders in each province **Medium quality**	+	+ rationalization of services	/	− organizational capacity		+	
ON pre- and post-LHIN creation					/evidence-based decision-making			
Fooks & Hylmar (2015)	Before and after study using Health Quality Ontario, Ontario Health Quality Council reports (2005/06 vs. 2014) **Low quality**				+ hip and knee surgery wait time, access to primary care			
Ontario pre- and post-LHIN creation								
Uchimura et al. (2019)	Grounded theory; interviews with health leaders and providers in two LHINs **Medium quality**			+ improved planning for patient needs, ability to understand local population	+ organizing and supporting pathways			
Ontario pre- and post-LHIN creation								
*Direct Comparisons*								
Barker & Church (2022) Alberta AHS comparison to Ontario LHINs	Cross-sectional analytic study using Ontario Ministry of Finance, Alberta Health Services, and other reports (2008/09/10 vs. 2016/17/18/19 based on data availability) **Low quality**		LHINs associated with slightly lower spending comparing with AHSAHS was associated with lower admin costs but LHINs had greater decrease in spending	Similar shifts in spending on community-based services	LHINs were associated with lower avoidable hospital admissions, but larger improvements over time were observed in AHSAHS were associated with shorter ED wait times for admissionSimilar results for readmission rates LHINs associated with lower wait times for five key surgeries, CTs and MRIs Patient experience similar, with higher scores on some items in Alberta		Similar citizen engagement activities across provinces	AHS and LHINs both experienced government intrusions that limited organizations’ ability to be fully accountable

+: improvement.

/: no change.

−: worsening.

^a^Outcomes from Bergevin were extracted only from interviewee statements directly attributed to specific provinces’ regionalization efforts. General statements such as “there was consensus among interviewees” or “experts identified” that did not refer to specific provinces were not extracted.

^b^For admitted patients and both complex and non-complex patients not admitted.

^c^Performance on four indicators improved between 2007 and 2010 but then plateaued or worsened since: cataract, hip, and knee replacements provided within 182 days and CT scans provided within 28 days.

^d^For patients with mental health or substance abuse conditions.

^e^Noted to be likely due to Ministry budget controls rather than LHIN efficiencies.

^f^Engagement opportunities increased but quality and representation varied across LHINs.

### RHAs vs. local structures

Bergevin et al.’s^6^ qualitative results suggest that regionalized structures are associated with improved integration and coordination of services, though not uniformly, and this study only included an average of three health system leaders from each province. Consolidation and rationalization of services to reduce costs was thought to have been achieved in most RHAs according to these interviewees but impressions of cost savings were mixed. Most also thought RHAs were able to better focus on illness prevention and public health post-merger but only New Brunswick was seen as having an improved ability to standardize best practices. Qualitative impressions of ability to match resource use with population needs and public participation were positive, but no information was available on whether accountability was improved in RHAs.^6^

The impact of Ontario’s LHINs, which had far less authority over hospitals and other providers than did RHAs, was assessed in five studies, including quantitative (low quality) and qualitative (medium quality) studies. Results were mixed. Access measures tended to improve but readmissions and repeat emergency visits worsened^8–10^ (Table 3).

### Provincial systems vs. RHAs

With a province-wide healthcare delivery system, Alberta achieved a reduction in administrative costs^11,12^ to around 2.7% compared to 4.3% on average for other provinces in 2021/22,^2^ though Nova Scotia reported no change.^3^ Surgical waiting times were stable in one study^13^ shortly after Alberta’s merger. Areas showing improvement in clinical outcomes in Alberta tended to be those for which there had been focused province-wide efforts towards addressing specific priorities. Qualitative results suggested both Alberta and PEI succeeded in rationalizing services in their restructuring, and Alberta was thought to have improved ability to integrate and coordinate a broad range of health services along with a better focus on health promotion.

Provincial structures can improve integration of non-clinical support services (e.g., information technology and procurement; Table 2). Central procurement is used by all current single provincial models but has also been deployed by some provinces with RHAs. Alberta and PEI are the only provinces with single Electronic Medical Records (EMRs) for hospitals, but Newfoundland and Quebec have announced they are moving to a single hospital EMR.

A single provincial model can support provincial scale and spread of effective clinical innovations^14,15^ through provincial clinical networks. These have been implemented in Alberta and Nova Scotia and are planned in Saskatchewan and Newfoundland. British Columbia and New Brunswick also have a small number of provincial networks that work in partnership with RHAs.

### Healthcare delivery structures for primary care and Indigenous people

Until the recent announcement of an Alberta provincial primary care organization (about which few details have been provided), no other province has a formal (non-voluntary) system of primary care governance. Several provinces have voluntary arrangements between medical associations, healthcare delivery systems, and governments, particularly those providing direct funding for interdisciplinary teams. Family physicians operate as independent contractors, making each their own silo, and receive little direction or support (beyond payment) from provincial governments or healthcare delivery systems.

Given the very poor health outcomes and the challenges in healthcare access and integration of care for Indigenous people, two provinces have created First Nations health authorities to support and manage Indigenous healthcare. British Columbia is the most advanced, with the First Nations Health Authority created in 2013 to plan and deliver care for First Nations communities. Nova Scotia announced a similar structure in 2023, Tajikeimik, to act as the Mi’kmaw health authority. Other provinces usually have Indigenous advisory committees or structures (without decision-making authority) to guide the care and integration of Indigenous healthcare services that are embedded within their broader healthcare delivery systems.^16^ Governance and organization of care for Indigenous people is an important consideration, which has been discussed further elsewhere.^17^

### Weaknesses of all healthcare delivery structure models

No studies have evaluated the impact of regionalization or centralization on physicians, but in interviews with Canadian health system leaders, Bergevin et al.^6^ noted that there was very weak engagement of physicians within all healthcare delivery systems. Leaders emphasized that funding and accountability for physician services and medications—two of the three largest components of health budgets and important drivers of system costs—sit outside the control of healthcare delivery systems. Interviewees noted this as a substantial and consistent weakness of all models.

The qualitative studies for health regions and centralized systems noted insufficient clarity in roles and responsibilities between the macro and meso levels. This blurring of the role of government and governance of meso-level structures, through health ministries’ willingness to interfere at the meso level, has been longstanding,^18^ but appears to be worsening over time.

## Discussion

Despite much being written about the justification for different meso-level healthcare delivery structures in Canada, limited research has explored their benefits and limitations. Nearly all studies are of low quality, and none use a rigorous comparator. This is surprising since of the over $340B that Canada spends on healthcare, approximately $190B is channelled through healthcare delivery structures.

There appear to be several potential pragmatic advantages to a single provincial delivery model, including the ability to integrate non-clinical support services provincially, which can lead to significant cost savings, but not all provinces have taken advantage of this opportunity. Integrating these services can make it easier to have a single provincial EMR, provincial pathways and clinical guidelines, and consistent measurement provincially through consolidated information technology and analytics teams—all of which can enable a learning health system.^19^ Integrated data and analytics could improve accountability and a single health system could enable provincial scale and spread through clinical networks or similar structures. Although centralized systems are at times criticized as being bureaucratic and less responsive to local needs, this could be mitigated by devolving more decision-making to zones, while ensuring zones remain accountable for clinical outcomes and costs.

It is too early to draw conclusions about Ontario Health Teams and their potential impact. OHTs are meant to coordinate delivery of the full continuum of care to a defined patient population, with some similarities to Accountable Care Organizations (ACOs) in the United States. The goals of American ACOs are to increase integration, improve healthcare quality, and reduce costs and health disparities. Unlike the American ACOs, no financial risk sharing is planned in OHTs, and key performance indicators have yet to be developed. Many questions remain about what these teams will do and whether they will counterbalance the power and influence of hospitals.^20^ Ontario Health will need to ensure accountability and enable provincial measurement of these indicators, which could be challenging with disconnected information technology/data and analytics groups and multiple EMRs.

One observation from this study is that, regardless of the model chosen, healthcare delivery structures on their own are likely not enough to improve health outcomes. This is not surprising. Donabedian^21^ noted that to improve healthcare quality and outcomes, there needs to be a focus on both structure and process (i.e., the clinical processes performed in the healthcare setting).

### Evidence from international comparators

Our focus was on reviewing studies from Canada. The issue of centralization versus regionalization is also germane in the European context though health regions are often associated with a local municipality, limiting comparability.^22^ In terms of meso-level reform internationally, the most direct comparison to Canada is likely New Zealand, which moved in 2000 from a central health ministry purchaser of healthcare to a regionalized system with 20 District Health Boards that operated hospitals and community-based services.^23^ District Health Boards allocated funding for primary care through primary health organizations, and physicians received capitation funding. In 2011, New Zealand organized District Health Boards into four regions, and there was a greater expectation to work together to plan and deliver services.

Reviews of the District Health Board system^24–26^ revealed that it was overly complicated and fragmented. Many District Health Boards had poor control of costs and there were weak incentives for regional collaboration meaning limited pooling of resources. Variation in services offered and quality of care across District Health Boards caused widespread concern about inequity across the system, particularly for Maori people. In 2022, New Zealand disbanded the District Health Boards and replaced them with two national healthcare delivery structures: Te Whatu Ora (Health New Zealand) and Te Aka Whai Ora (Maori Health Authority).^27^ The goal of this reorganization is addressing inequitable access to health services and health outcomes.^28^ New primary care arrangements have yet to be announced but Primary Health Organizations will no longer exist, in part given the complexity of relationships between District Health Boards and primary health organizations.^29^

## Recommendations

It is unfortunate that nearly a decade after Bergevin et al.’s review of regionalization,^6^ the seven areas for improvement identified in that report, paraphrased below, are still sorely in need of attention today:

• Manage healthcare delivery systems as results-driven health programs transforming them into high performing health systems;
• Strengthen wellness promotion, public health, and intersectoral action for health to better address social determinants of health;
• Ensure timely access to better primary healthcare;
• Involve physicians in health system leadership, pay them through (and make them accountable to) the health system, and have physicians and health systems co-accountable for system results;
• Engage citizens in shaping their own health destiny and their health system;
• Strengthen health information systems, make them interoperable, and use them to measure indicators that reflect health system priorities;
• Build a learning health system by fostering a culture of excellence, learning, innovation, and continuous quality improvement.

Despite significant structural changes in recent years, most provinces have not addressed the broader problems that exist in Canadian healthcare which influence healthcare delivery systems’ ability to achieve the Quadruple Aim. These include a lack of investment in the broader determinants of health, the lack of a long-term plan for healthcare systems, no organizational structure and not enough funding for primary care, physician-related challenges including payment models, and a lack of accountability for physicians and healthcare delivery systems.

With respect to healthcare delivery system restructuring, it needs to be done with the explicit goals of improving the Quadruple Aim in mind, with concurrent evaluation and a plan for refinement built into any restructuring. Patient and public engagement needs to be built into all stages of planning for and evaluation of health system restructuring. Healthcare delivery systems need to create healthcare processes and ensure a system exists to scale and spread healthcare improvements provincially. Finally, there needs to be more attention to clarifying roles and responsibilities between the healthcare delivery system and health ministries/governments.

It is surprising how little evidence, particularly on clinical outcomes and overall healthcare costs, is available to inform the choice of healthcare delivery models. These research gaps could be addressed using rigorous study designs and data from the Canadian Institutes of Health Information (which includes measures of health outcomes, patient experience, and costs), given that differences in provincial healthcare delivery structures have existed for over 15 years. These studies would need to control for other health system differences (e.g., physician payment models and changes in healthcare budgets), and there would be a role for qualitative studies including health system leaders, staff, physicians, and patients. Given ongoing major structural changes in healthcare delivery systems across Canada, we recommend that the Canadian Institutes of Health Research launch a special funding competition to determine their differential impact.

### Limitations

As noted elsewhere, there is a dearth of high-quality research on which to base strong conclusions. While we excluded commentaries, news articles, and reviews without data from our study, we were still left with only low quality quantitative papers and medium quality qualitative papers. Our literature search found no controlled rigorous study designs with appropriate comparators. There were few studies directly assessing aspects of the Quadruple Aim, or the impact of restructuring on staff or physicians. Finally, no studies assessed potential detrimental effects of reorganization on staff experience and patient outcomes during the transition period.

## Conclusion

There is significant variation across Canada in provincial structures for healthcare delivery. Many provinces have been shifting towards a single healthcare delivery system, though Alberta is now moving away from this without a clear understanding of how changes will influence outcomes or costs. The pros and cons of this restructuring, which has not been tested elsewhere, remain to be determined. While we may not be able to draw firm conclusions about the optimal structure based on the low quality of evidence currently available, major transformations are highly disruptive and costly for health systems, and reorganization cannot fix many of the problems that exist in Canadian healthcare more broadly.

Based on the current evidence, a meso-level provincial organization, separate from the health ministry to enable longer-range health services planning, and with embedded zones that are accountable to the provincial structure, would appear to hold the most promise based on the capabilities that such a system can enable. However, regardless of the structure chosen, it is clear that no provinces have optimized their processes to achieve consistent high-quality outcomes at a reasonable cost. More support, planning, and organization are required for primary care. Work is also required to improve system integration, and to involve physicians more fully within the healthcare system (which will require major changes to payment models) to reduce care variation.^3,6–13,30^
